# Effect of Skull Type on the Relative Size of Cerebral Cortex and Lateral Ventricles in Dogs

**DOI:** 10.3389/fvets.2017.00030

**Published:** 2017-03-16

**Authors:** Anders M. Pilegaard, Mette Berendt, Pernille Holst, Arne Møller, Fintan J. McEvoy

**Affiliations:** ^1^Department of Veterinary Clinical and Animal Sciences, University of Copenhagen, Frederiksberg, Denmark; ^2^Center of Functionally Integrative Neuroscience, Department of Clinical Medicine, Aarhus University, Aarhus, Denmark

**Keywords:** canine cognitive dysfunction, hydrocephalus, skull type, lateral ventricles, cerebrum, cavalieri’s principle

## Abstract

Volume measurements of the brain are of interest in the diagnosis of brain pathology. This is particularly so in the investigation hydrocephalus and canine cognitive dysfunction (CCD), both of which result in thinning of the cerebral cortex and enlarged ventricles. Volume assessment can be made using computed tomography or more usually magnetic resonance imaging (MRI). There is, however, some uncertainty in the interpretation of such volume data due to the great variation in skull size and shape seen in dog. In this retrospective study, we examined normal MRI images from 63 dogs <6 years of age. We used a continuous variable, the cranial index (CrI) to indicate skull shape and compared it with MRI volume measurements derived using Cavalieri’s principle. We found a negative correlation between CrI and the ratio of cortical to ventricular volume. Breeds with a high CrI (large laterolateral compared to rostrocaudal cranial cavity dimension) had a smaller ratio of cortical to ventricular volume (low C:V ratio) than breeds with lower CrI skull types. It is important to consider this effect of skull shape on the relative volume estimates of the cerebral cortex and ventricles when trying to establish if pathology is present.

## 1 Introduction

Measurements of cerebral cortical and ventricular volumes are of diagnostic interest in the dog. This is especially so in dogs suspected of canine cognitive dysfunction (CCD). This is a disease of elderly dogs (≥8 years) resembling human Alzheimer’s disease (1, 2). CCD is macroscopically characterized by cortical atrophy and widening of the ventricles and immunohistochemically by, e.g., deposition of the peptide, amyloid beta (*Aβ*) (3–10). Parameters that describes cerebral and ventricular volumes, such as the ratio of the volume of cerebral cortex to that of the lateral ventricles (C:V ratio) are of interest when trying to establish an antemortem diagnosis of CCD. Volume data alone cannot make such a diagnosis, but these data can be combined with clinical examination findings, including cognitive tests to help diagnostic accuracy. Various methods have been used to estimate the volume of brain structures on MRI scans in dogs. Each method has its own advantages and disadvantages. Visual rating scales are open to bias and subjectivity and yield a categorical variable but have been used for evaluating brain atrophy in aging dogs (7). Others have used the interthalamic adhesion thickness (5), which is a simple measurement to perform but may not accurately reflect three-dimensional structures. In the diagnosis of CCD, Cavalieri’s principle was used to demonstrate a significantly smaller cortex/ventricle ratio when comparing a group of dogs with CCD (mean age, 12.6 years) with a healthy control group (mean age, 8.8 years) (11). It is uncertain to what extent differences in the C:V ratio relate to pathology or to breed differences in skull size and shape. It is these differences in dogs without CCD or other disease known to influence brain volume that this study addresses.

Skull shape in dogs is commonly classified as dolichocephalic, mesaticephalic, or brachycephalic (12). An alternative approach to this categorical variable for classification is to use a continuous variable. Such a variable can be constructed as an index derived from various skull dimensions. One such index has been designated the cranial index (CrI) (12). This index can be used not only in analysis as a continuous variable but also to categorize dogs as brachycephalic (high CrI), mesaticephalic (intermediate CrI), or dolichocephalic (low CrI). Studies on the influence of skull shape on the regions of the brain, e.g., the olfactory bulb (13) and ventricles (14, 15), indicate that the size of the cerebral ventricles might be correlated to skull shape. The classification of skull shape is not uniform across studies.

In this retrospective study, we use Cavalieri’s principle to determine ventricular and cerebral volumes in dogs of different breeds and size. Cavalieri’s principle is an established methodology in volumetric stereological measurement. It is particularly easily applied to MRI, computer tomography, and Positron emission tomography images of brain structures (16–20). The measurements obtained are used to calculate the C:V ratio. We examine the relationship between this ratio and an index of skull shape to address the hypothesis that skull shape has and effect on C:V ratio in dogs. We further examined the reproducibility of our measurements to give an indication of clinical applicability.

## 2 Materials and Methods

### 2.1 Study Population

Records for dogs ≤6 years old that had MRI scans performed as part of a clinical investigation for neurological disease at the Copenhagen University Hospital for Companion Animals, Denmark, between 2008 and 2012 were reviewed. Inclusion criteria required that the images were evaluated by a board-certified radiologist and were found to be without imaging evidence of pathology.

### 2.2 Imaging and Randomization

Images were obtained using a low-field MRI scanner [Esaote Vet-scan 0.2 Tesla (Esaote Group, Genova, Italy)]. All dogs were placed under general anesthesia for imaging. MRI images were viewed and measured in a DICOM viewer (RemotEye, NeoLogica s.r.l., Cairo Montenotte, Italy). Only T1-weighted images (TE in the range 16.00–18.00 ms and TR 600–800) in transverse and dorsal planes were used for evaluation of cortical and lateral ventricular size in this study. MRI scan measurements were made by the first author (AP) after training from a board-certified specialist in veterinary radiology (FM) and a medical neurologist (AM) in the use of the medical viewer, recognition of relevant brain anatomy, assessment of the cranial index (CrI), and the application of Cavalieri’s principle. Patients were randomized for MRI measurements.

### 2.3 Cranial Index

The cranial index (CrI) was used as a descriptor of skull shape (12). It describes the width of the skull in relation to its length. CrI was calculated as follows:

(1) $$CrI=\frac{Cranial\;width}{Cranial\;length}\cdot 100.$$

The cranial length is defined as being the maximum distance from the cranial to the caudal aspect of the cranial cavity. The cranial width was defined as the maximum distance (lateral to lateral) across the cranial cavity. These distances were measured from sagittal (cranial length) and transverse (cranial width) plane MRI images.

### 2.4 Cavalieri’s Principle

Volumetric estimation of cortex size and the lateral ventricle size was achieved using Cavalieri’s principle. This stereological method allows estimation of volume as follows:

(2) $$V_{Total}=d\cdot a_{p}\cdot \sum p,$$

where *V_Total_* is the total volume of the object to be measured, *d* is the distance between the sections that are being analyzed (i.e., slice thickness plus slice interval), *a_p_* is the area associated with one counting point (see below), and ∑*p* is the sum of all such points counted for the given object, in this case either the cortex or ventricle. Because of contrast resolution limitations imposed by the magnetic field strength, cortex was defined as both white and gray substance. Structures not included as cortex included the olfactory bulb, hippocampus, pons, brain stem, thalamus, cerebellum, and structures associated with these. To ensure unbiased estimations, Cavalieri’s principle requires that the object to be measured is placed randomly with respect to the grid and that the entire object is included in the image stack. In MRI scanning, the former is easily achieved since the first section (slice) of a sequence is randomly positioned within the patient. There is no specific alignment of any anatomical feature on the z-axis of the scanner, so that the start point is essentially random.

#### 2.4.1 Counting Procedure

**Figure 1:**
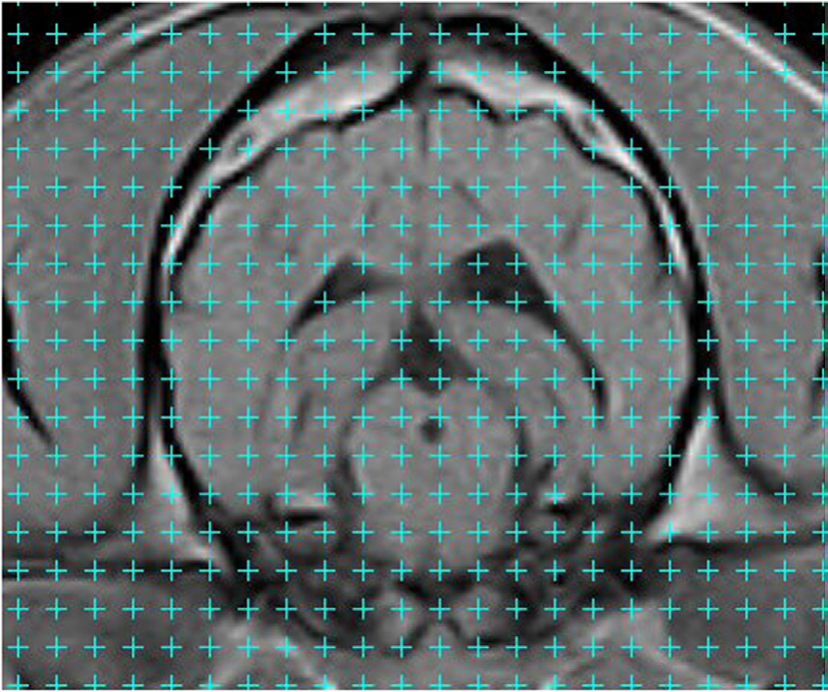
**Transverse T1-weighted magnetic resonance image of the cranial cavity taken at the level of the temporomandibular joints**. A grid pattern has been applied. Volume measurement is achieved by counting the number of counting points superimposed on the structure to be measured. Counting points are located at the upper right corner of each of the crosses in the grid (i.e., for each cross, adjacent to the intersection of the two cross lines, at the pixel that is immediately right of the vertical line and immediately above the horizontal line of the cross). The counting process is a series of yes:no questions, most of which are readily answered.

**Figure 2:**
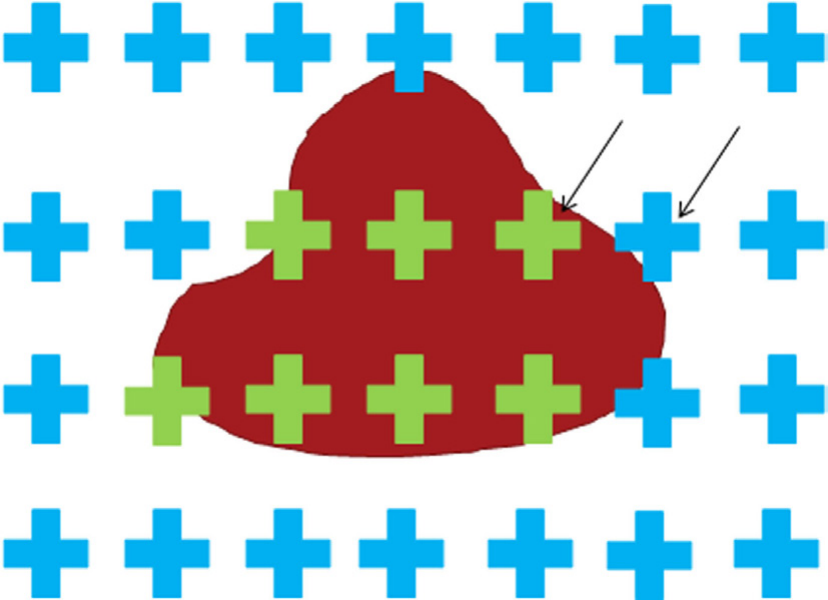
**Schematic of the point counting technique**. The crosses serve to help the reader identify the counting points that are located at the upper right corner of each cross. The black arrows indicate two such points. The only decision to be made is whether the counting point is or is not on the object. In this illustration, the blue color indicates that the point associated with the cross does not intersect the object. The green color indicates that the point associated with the cross does intersect the object.

The first author (AP) performed the counting procedure. Two image planes were used in the counting procedure: the sagittal plane images to assist the orientation of brain structures and transverse plane images for actual counting. The cortex and ventricles were measured separately. The counting procedure starts at the most rostral slice that includes the structure (cortex or ventricle) to be measured. Counting continues caudally to the most caudal slice that includes the structure to be measured. Counting requires the use of a grid, with regularly spaced crosses, which is superimposed on the image. This can be generated digitally and superimposed on the image if the viewing software permits or, as was done for this study, can be printed on a transparent sheet of plastic (A4 size) and placed directly on the viewing monitor, superimposed on the sections to be measured. Once placed on the monitor, the grid is left *in situ* for the entire counting procedure. Slice thickness was set in this study in the range 4.5–5 mm. By scrolling through the stack of transverse images, a check was made to ensure that images were zoomed to allow satisfactory evaluation of each structure to be measured (ventricle or cortex) but not so enlarged that any part of the structure extend beyond the limits of point counting grid. Once a suitable level of zoom was established for the particular region (ventricle or cortex), it was maintained for evaluation of that region in the entire stack in that patient. The image distance represented by the distance between the counting points on the grid for that particular image stack and zoom setting was then measured using the viewer software. The square of this distance gives the area associated with each counting point, *a_p_* for the variable in equation (2). The degree of zooming was unchanged within any region, ventricle or cortex in a particular individual, but it can be and typically was altered between regions and individuals. Thus, values for *a_p_* are specific to the individual patient and the region examined. The act of counting requires that the reader determines how many grid counting points intersect the structure being measured. It amounts to a series of yes, no decisions. An example of the grid arrangement is shown in Figure 1, and a schematic for the counting procedure is shown in Figure 2. The CE for the measurements was calculated as described below using methodology based on previously described techniques (17).

### 2.5 Measurement Reliability

#### 2.5.1 Interrater Agreement

A second evaluator (PH) was enlisted for determining interrater agreement for volume estimates. The training session for this individual comprised instruction in identifying brain structures, measuring cranial length and width, and performing the counting procedure. Ten random image sets were used for training. This same individual then performed the measurements in 9 other dogs from the study population that were also chosen randomly for the inter–rater analyses. These values were then compared with those obtained in a repeat analysis in the same 9 individuals by the other evaluator (AP). Both evaluators were blinded to the results of previous measurements.

#### 2.5.2 Intrarater Agreement

The same 9 dogs mentioned above for interrater analysis were also used for the intrarater reliability testing. The same individual (AP) performed repeated measurements at least 2 weeks after the initial measurements. This ensured that individual image stacks from the initial reading were not recalled during the second measurement session.

### 2.6 Data Analysis

CrI measurements for each dog were plotted against log-transformed C:V ratio. Correlation between CrI and the transformed C:V ratio was tested using Spearman’s rank correlation coefficient (a non-Gaussian distribution was assumed). Linear regression was also used to test linearity of the relationship between the two variables.

Two possible confounding factors, age and weight, were tested against C:V ratio and CrI individually, to reveal possible associations, using the same methods as described above. For evaluation of interrater and intrarater agreement, Bland–Altman difference plots were used. All statistical data analysis was done using either the statistical software GraphPad Prism v.6 (GraphPad Software Inc., La Jolla, CA, USA) or R (version 3.3.1, R Foundation for Statistical Computing, Vienna, Austria) (21) according to availability. A *p* value <0.05 was considered statistically significant.

## 3 Results

### 3.1 Study Population

Sixty–three dogs met the inclusion criteria. Scans were performed for a variety of reasons mainly for the investigation of epilepsy (47 dogs), seizures (6 dogs), and lesser numbers for investigation of other suspected diseases including middle ear disease. The mean age was 2.5 years (median, 2 years; SD, 1.6 years). Mean body weight was 20.8 kg (median, 18 kg; SD, 14.4 kg). A total of 40 dog breeds were included. Breeds encountered were retriever type dogs (n = 8), followed by Cavalier King Charles spaniels (n = 4) and lesser numbers of the other breeds.

### 3.2 Cortex: Ventricular Ratio (C:V Ratio) and Cranial Index (CrI) Estimates

**Figure 3:**
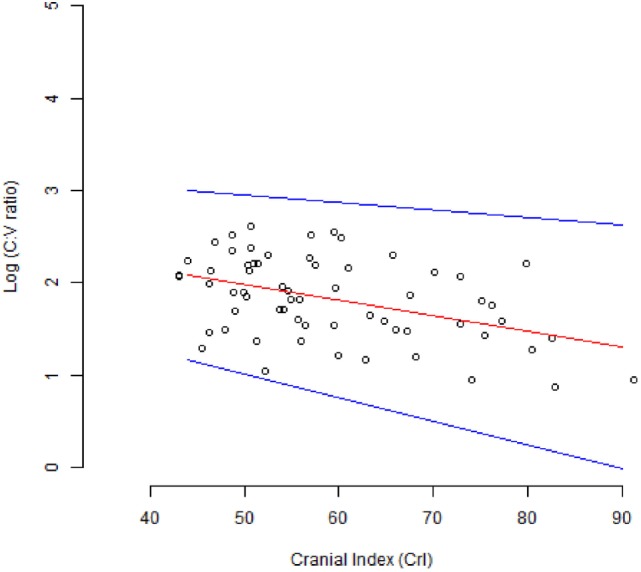
**Scatter plot of the relationship between the *log*_10_*C*: *V ratio* and the cranial index (CrI)**. The red line shows the line of best fit for the linear regression model. The blue lines show the 95% confidence interval.

Volume estimates of the cortex ranged from 21.8 to 77.8 cm^3^, while those for the ventricle ranged from 0.2 to 4.6 cm^3^. This resulted in a range in C:V ratio values from 7.5 to 408. Values of CrI ranged from 43 to 91%. There was a significant negative correlation between CrI and *log*_10_ C:V ratio (r = −0.44, *p* = 0.0003, 95%confidence interval = −0.62 to −0.21). A linear regression model for the association between CrI and *log*_10_ C:V ratio showed the relationship between the variables to be represented by the equation *CrI* = 2.8 − 0.02log_10_(*C*: *V ratio*). This regression line is shown in Figure 3 together with its 95% confidence interval.

### 3.3 Measurement Reliability

#### 3.3.1 Interrater Agreement

The interrater agreement for C:V ratio was 82%. This result and other interrater agreement data are given in Table 1. The variability between readers was independent of the size of measurements concerned.

**Table 1:** **Intrarater and interrater agreement for measurements made from 9 samples using Cavalieri’s principle**.

	Bias (agreement%)	95% limits of agreement
**Intrarater agreement**		
C:V ratio	6 (94)	−43 to 55
Cortical volume	3 (97)	−15 to 21
Ventricular volume	22.9 (77.1)	−48 to 41.7
CrI	0.1 (99.9)	−8 to 8
**Interrater agreement**		
C:V ratio	−18 (82)	−81 to 46
Cortical volume	−2 (98)	−23 to 18
Ventricular volume	15 (85)	−49 to 79
CrI	4 (96)	−48 to 79

*Dimensions for bias are *mm*^3^ for both cortical and ventricular volumes. Bias values for cortex to ventricular volume ratio (C:V ratio) and for cranial index (CrI) are dimensionless. Agreement is quoted as percent*.

#### 3.3.2 Intrarater Reliability

The intrarater agreement for C:V ratio was 94%. This result and other intrarater agreement data are also given in Table 1. The within-reader variability was independent of the size of measurements concerned.

### 3.4 Confounding Factors

No associations were found between weight, age, and C:V ratio nor between age and CrI. A negative linear correlation (r = 0.74, *p* < 0.0001) was seen between weight and CrI. These data, however, also showed a significant deviation from linearity (*p* = 0.0035). Thus, confirmation of an association between weight and CrI would require non-linear regression, which was beyond the scope of this study.

## 4 Discussion

The results show a significant moderate negative correlation, r = −0.44 (*p* = 0.0003), between the log transform of the C:V ratio and CrI, the latter used as a measurement of skull type. The log transform of the volume measurements resulted in a correlation that was more readily observed and that could be represented by a simple linear model. The linear regression is shown in Figure 3. As the CrI of the dog skull increased, the C:V ratio decreased. This decrease in C:V ratio could be explained by a naturally larger ventricular volume, a smaller cortical volume, or a combination of the two. This is likely a physiological or breed-related phenomenon rather than CCD-associated cortical atrophy, since all of the dogs participating were ≤6 years of age and too young to show changes of CCD.

Breed-related differences in ventricle size would explain our findings and accord with findings from other studies. In one study, miniature and brachycephalic breeds in particular exhibited what was defined as ventriculomegaly (15). The same study showed no correlation between ventricular size and clinical signs of neurological disease. In another study, MRI images from Yorkshire Terriers were compared to images from German Shepherd dogs (14). A significant difference in the ratio of ventricular size to cortex between the two breeds was found, and again there were no findings to indicate that the variations in ventricular size demonstrated were associated with neurological disease. These results indicate that there is a breed-related variation in ventricular size.

We chose to use a continuous variable, the CrI, as a descriptor of cranial shape. The alternative of dividing dogs into categories depending on skull type (brachycephalic, mesaticephalic, or dolichocephalic) is problematic (14). First, the division on the basis of breed is unreliable, as within-breed variation of skull type can be significant. In-breed variation in ventricle size has been demonstrated, and this could be linked to in-breed variation of skull type (22). Second, no clear and generally agreed criteria exist for placement of a breed in a particular category. There are differing criteria concerning methods of measurements and thresholds for the three categories mentioned above. The criteria chosen may vary between studies. In three separate studies, dogs of the boxer breed were assigned to the brachycephalic category in one study, mesaticephalic category in a second study, and the dolichocephalic category in third study (12, 23, 24). Finally, the three categories are arbitrary designations and are not based on clinical considerations. A continuous scale of measurement is more desirable, and thus, CrI was used in this study.

Analysis of possible confounding factors in this study found a negative correlation between CrI and body weight. This association could be explained by the fact that many dogs with high CrI were from the smaller breeds, which have been selected for a brachycephalic type head conformation.

The method of volume estimation utilizes Cavalieri’s principle. This volumetric stereological measurement technique has been used for estimation of brain volume and recommended for clinical use in elderly people with dementia (25). The method has been used also in the evaluation of tumor volume from MRI images and again recommended for clinical use (26). The counting procedure lasted on average approximately 10 minutes per patient. An advantage of this technique is that the position of most counting points, cortical or ventricular, will be clear. Only a few will be on a border between the two regions and require effort for a decision. In contrast methods that require the operator to outline the limits of a structure by definition require the user to constantly work at the image area of maximum uncertainty; the border between two tissues. An additional benefit is that the coefficient of error (CE) for the method can be calculated. This gives a statistical estimate of the reliability of the measurements and depends in part on the relationship between the grid spacing and the object to be measured. The greater the number of grid point counts, the lower the CE. The CE is also dependent on the number of slices encompassing the object to be measured. This is a function of object size and slice thickness. It is accepted that while the ease with which measurements can be made and volumes calculated are merits of the technique; it is also true that the calculation of the coefficient of error is somewhat involved.

There are many alternative methods for evaluating the volume of body structures, and these include, but are not limited to, manual or automatic segmentation methods that require the creation of regions of interest. Region growing algorithms and other methods for determining volumes from image stacks are available in widely used software such as ImageJ (27) and Osirix (28). However, their use for this purpose is typically time consuming when evaluating structures that are irregular and complicated in shape and will result in volume estimates with uncertain error coefficients. Also, the structure to be measured must completely contrast with adjacent tissue, otherwise the region of interest will escape the structure to be measured. Methodologies such as measurement of interthalamic adhesion measurement are simple to perform but are oversimplistic in that it is difficult for a one-dimension spatial measurement to represent a geometrically irregular volume. In contrast, the methodology used in this article lacks the apparent sophistication of analysis software, is simple to perform, and yet provides a volume estimate, together with its coefficient of error. It is a methodology that is not widely used in the veterinary community but perhaps deserves more attention.

Our results show excellent interrater and intrarater agreement with the method. The low coefficient of error (3.4%) in cortical estimations and the high levels of intrarater and interrater agreement (97 and 98%, respectively) show that the method has high precision when used to estimate cortex volume in a dog brain. Measurements of the ventricles were less precise (coefficient of error 13%) due to their smaller volume. The higher coefficient of error values was obtained from scans in which the ventricles were visible on either 3 or on 4 slices only. Volume estimates from low numbers of slices will result in a high coefficient of error. The error in the method, however, is symmetrically distributed, so that one is as likely to overestimate a volume as to underestimate it. Therefore, we do not think that there is a bias in this regard. Compensating for low slice numbers by selecting a smaller point grid and thus more counts per slice will help reduce the coefficient of error, but only to a limited extent and at the cost of disproportionate increased effort in point counting. The method ideally requires that the object to be measured appears on 6–8 slices or more. Slice thickness should be chosen accordingly. An MRI scanner with a higher magnetic field strength would have allowed thinner and therefore more slices with superior contrast resolution than those available from the low-field magnet used in this study.

Notwithstanding these reservations, the C:V ratio appears to be a good candidate for assisting in the diagnosis of conditions such as CCD, where cortical and ventricular volumes are important. The results of this study suggest that a correction based on the CrI can be applied for comparisons between dogs of different breed. If such a correction step is not taken, a low C:V ratio in breeds with a high CrI may be misinterpreted. This study comprised 63 dogs. Future experience with a with a larger cohort of healthy young dogs would of course assist in validating and defining this negative correlation between C:V ratio and CrI. Ultimately volume measurements will have to prove their clinical relevance for diagnosis or prognosis. The effect of aging on these indices also requires evaluation since age-related change in the ventricular system in clinically healthy dogs has been described (4, 6), and this should be differentiated from changes potentially related to disease.

## 5 Conclusion

This study shows an association between the ratio of cortical to ventricular volume (C:V ratio) and skull type, represented by CrI. Breeds with a high CrI (large latero-lateral compared to rostro-caudal cranial cavity dimension) have a smaller ratio of cortical to ventricular volume (low C:V ratio) in comparison to breeds with lower CrI skull types. The index, C:V ratio, is a good candidate for future evaluation of cortical atrophy and ventricular dilation in elderly dogs and in dogs with CCD. In addition, this application of Cavalieri’s principle to canine brain measurements resulted in high intrarater and interrater agreement. The simplicity of the method and the lack of special software requirements make it attractive for both the clinical researcher and general practitioner.

## 6 Equations

### Coefficient of Error Calculations

Let the series of measurements in *n* slices that intersect a structure be denoted by *P*_1_, *P*_2_, …, *P_n_*, so that the number of points intersecting the structure in the *i*th slice is *P_i_*. If *A, B*, and *C* are defined such that

$$A=\sum_{i=1}^{n}\;P_{i}\cdot P_{i}B=\sum_{i=1}^{n-1}\;P_{i}\cdot P_{i+1}C=\sum_{i=1}^{n-2}\;P_{i}\cdot P_{i+2},$$

then the variance due to systematic sampling and measurement error, *Var_sys_* is given by:

$$Var_{sys}=\frac{1}{240}\cdot (3\cdot (A-Var_{noise})-4\cdot B+C).$$

The variance due to noise, *Var_noise_* is given by:

$$Var_{noise}=0.0724\cdot 8\cdot \sqrt{\sum_{i=1}^{n}\;n\cdot P_{i}},$$

where the constant 0.0724 is derived from the Riemann zeta function and the constant 8 is the cube of a form factor of 2, which has been chosen based on experience with the technique in brain volume analysis.

The total coefficient of error *CE* for the volume measurement can be calculated:

$$CE=\frac{\sqrt{Var_{sys}+Var_{noise}}}{\sum_{i=1}^{n}P_{i}\;}.$$

## Ethics Statement

This retrospective study used imaging data acquired from veterinary patients at the university teaching hospital. As such these were animals under veterinary care and were not designated “experimental animals.”

## Author Contributions

AP, MB, PH, and FM made substantial contributions to the design of the study, data acquisition, analysis, and interpretation. AM made substantial contributions to the design of the study, analysis, and interpretation.

## Conflict of Interest Statement

The authors declare that the research was conducted in the absence of any commercial or financial relationships that could be construed as a potential conflict of interest.
